# Respiratory Syncytial Virus (RSV) in an Italian Pediatric Cohort: Genomic Analysis and Circulation Pattern in the Season 2022–2023

**DOI:** 10.1002/jmv.70660

**Published:** 2025-10-23

**Authors:** Alessia Lai, Annalisa Bergna, Valentina Fabiano, Carla della Ventura, Hanin Chmes, Alice Romero, Martina Loiodice, Angela Maria Dolores Boria, Alessandro Campagnoli, Felicia Stefania Falvella, Alberto Dolci, Gian Vincenzo Zuccotti, Gianguglielmo Zehender

**Affiliations:** ^1^ Department of Biomedical and Clinical Sciences University of Milan Milan Italy; ^2^ Laboratory of Medical Microbiology and Virology University of Insubria Varese Italy; ^3^ Paediatric Department “Vittore Buzzi” Children′s Hospital Milan Italy; ^4^ SC Patologia Clinica ASST Fatebenefratelli‐Sacco Milan Italy

**Keywords:** genomic epidemiology, pediatric cohort, respiratory syncytial virus, whole‐genome sequencing

## Abstract

There is growing interest in the molecular surveillance of the respiratory syncytial virus (RSV) and data concerning the virus molecular epidemiology in high‐risk pediatric patients in Italy are still limited. A total of 127 RSV‐positive swabs collected in 2022–2023 season were analyzed. Whole genomes were obtained by next‐generation sequencing and used for phylogenetic and phylodynamic analyses. A large proportion of the subjects had required hospitalization (78%) and age of hospitalized subjects was significantly lower than that of nonhospitalized (69 vs. 129 days; *p* < 0.0001). Genomic analysis suggested a significant increase in nucleotide variability in recent samples compared to previous waves and especially in subtype A. Phylogenetic analysis identified 14 and 16 clades including Italian strains in RSV‐A and B, respectively. Italian strains tended to group together forming monophyletic groups, 9 in RSV‐A and 13 in RSV‐B, probably representing local chains of transmission. A few of those pure Italian subclades began in 21–22 wave and persisted for more than 1 year. For both subtypes the skyline plot showed two peaks in transmissions, the first between 2017 and 2019, followed by a temporary reduction in 2021 coinciding with the widespread use of control measures against COVID‐19 and the second at the beginning of 2023. Accordingly, the Re estimates showed fluctuating values. This study suggests that the large circulation of RSV following pandemic restrictions is partly due to the introduction of viral strains already circulating across Europe, and partly to strains that persist in our region from one season to the next.

## 1 Background

Human respiratory syncytial virus (HRSV) is a common viral pathogen that causes around 64 million acute respiratory infections annually and is considered as a major cause of lower respiratory tract infections among young children, elderly, and immunocompromised adults worldwide [1].

In 2019, HRSV was associated with more than half of infections and hospital admissions in children aged less than 2 years and was responsible of around one in every 50 deaths in children aged 0–60 months and one in every 28 deaths in children aged 28 days to 6 months [2, 3]

Since the start of the reporting period for respiratory viruses and up to week 47/2022, 23 EU/EEA countries to The European Surveillance System reported 25 838 from 244 325 specimens′ positivity RSV (10.6%) [4]. Consequently, RSV infections take place a considerable role on healthcare systems and the global economy, and impact on young children′s health [5].

RSV is an enveloped virus with a single‐stranded negative‐sense RNA genome of approximately 15.2 kb that encodes 11 proteins. The glycoprotein G and fusion protein F are essential for entry of the virus into host cells. RSV encompasses two main subtypes, RSV‐A and RSV‐B [6], which are further divided into several genotypes based on genetic differences in hypervariable region 2 (HVR2) of the G protein [7].

However, since 2020, according to samples published in the GISAID (Global Initiative on Sharing All Influenza Data) [8] database, over 98% of RSV‐A isolates belong to genotype GA2.3.5, which includes in the G protein the insertion 261–284 and all RSV‐B belong to genotype 5.0.5a, with insertion 245–264 in the G protein.

Nonetheless, other areas of the RSV genome, although less variable, are also relevant for surveillance purposes. Recently, two prevention strategies, both targeting F gene, became available for protecting infants against RSV infection, including passive immunization of young infants through vaccination of pregnant women (maternal immunization) and administration of long‐acting monoclonal antibodies (mAbs) such as nirsevimab to neonates and infants. The F gene should therefore be checked for mutations that could influence the effectiveness of prophylaxis [9, 10, 11].

Currently, RSV is not an officially notifiable infectious disease in Italy. However, European Centre for Disease Prevention and Control (ECDC) recently recommended as a priority for the European Union and the World Health Organization integrated surveillance of respiratory viruses (including influenza, severe acute respiratory syndrome coronavirus 2, and RSV in addition to other important respiratory viruses) based on sentinel systems in primary and secondary care. In Italy, RSV surveillance has been implemented as part of the influenza‐like illness surveillance network [12], and some European countries have reported data to the ECDC allowing assessment of RSV activity in Europe in recent years.

As next‐generation sequencing (NGS) has become more available and affordable, whole‐genome sequencing (WGS) has become a common tool in epidemiological surveillance following the pandemic. In addition, the integration of RSV data into the GISAID database and Nextstrain platform [13, 14] is contributing to a greater understanding of RSV variability and spread globally.

A recent Italian study described a seasonal alternation of RSV‐A and RSV‐B serotypes in hospitalized‐cases between 2015 and 2018 [15]. Globally, in Italy subtype B was predominant until 2018, with some differences observed between northern and southern Italy [16, 17]. Particularly, more recent data on the genomic epidemiology of RSV showed an equal proportion of RSV‐A and RSV‐B during the 2021–2022 season in Northern Italy [18].

At present, data concerning the molecular epidemiological characteristics of RSV subtypes in high‐risk pediatric patients in Italy are still limited. In this study, we present the WGS and the genomic epidemiology of the main variants of RSV circulating in Italy during the 2022–2023 season in an international context, and in comparison, with previous seasons.

## 2 Materials and Methods

### 2.1 Specimen Collection

A total of 127 nasopharyngeal swabs resulted positive for RSV infection were collected from the Vittore Buzzi Children′s Hospital in Milan between December 2022 and March 2023. No additional samples were required, and those analyzed were obtained using leftover samples of diagnostic testing stored at −80°C and shipped every 2 weeks. For all participants informed consent was obtained from the parents or legal guardians. All subjects were enrolled before the introduction of nirsevimab prophylaxis in Italy, thereby reflecting the pre‐prophylaxis landscape. All data used in this study were previously anonymized as required by the Italian Data Protection Code (Legislative Decree 196/2003) and the general authorizations issued by the Data Protection Authority. The study was conducted in compliance with Good Clinical Practice and the Declaration of Helsinki.

### 2.2 Subtype Assignment and Whole Genome Amplification

Viral RNA was extracted with QIAamp Viral RNA Mini Kit 116 (QIAGEN, Hilden, Germany) following manufacturer′s instructions and eluted in a final volume of 50 μL of water. The RSV subtype was determined by reverse transcription polymerase chain reaction (RT‐PCR) as previously described [18]. To synthesize first‐strand cDNA 10 μL of RNA was added to 4 μL of LunaScript® RT SuperMix Kit (New England BioLabs, Ipswich, MA, USA) and 6 μL of nuclease‐free water.

Thermocycling conditions consisted of 2 min at 25°C, 55°C for 20 min, and 98°C for 1 min following manufacturer′s instructions. Only samples with Ct less than 30 were subsequently amplified.

Two distinct amplification protocols were applied for RSV‐A or RSV‐B.

For RSV‐A the design of primers was built as previously published work [18]. In summary, 40 primers divided into two pools were used (Supporting Information: Table S1), with each pool covering all the genome using an amplicon length ranging from 1014 to 834 bp. Two PCR reactions were performed in a 25‐μL reaction system containing 6 μL of template RNA, 3.6 μL of each primer pool diluted to 10 μM for reaction, 12.5 μL of Q5® Hot Start High‐Fidelity 2X Master Mix (New England BioLabs, MA, USA), and 2.9 μL of nuclease‐free water. Thermocycling conditions consisted of 98°C for 30 s, followed by 35 cycles of 95°C for 15 s, and 62°C for 5 min.

For RSV‐B a similar approach was applied using primalscheme [19, 20] (Supporting Information: Table S2) and an amplicon length of 400 bp. PCR reactions were modified as follows: 4 μL of template RNA, 2.5 μL of each primer pool diluted to 10 μM for reaction, 12.5 μL of Q5® Hot Start High‐Fidelity 2X Master Mix (New England BioLabs, MA, USA), and 4 μL of nuclease‐free water for a total volume of 25 μL. The same thermocycling conditions of RSV‐A were used.

### 2.3 Whole‐Genome Sequencing

Pooled amplicons were purified using Agencourt AMPure XP Beads (Beckman Coulter, CA, USA) and checked on a 4200 TapeStation System (Agilent Technologies, Santa Clara, CA, USA). Library concentration was determined with the Invitrogen Quant‐iT Picogreen dsDNA assay (Fisher Thermo Scientific, Waltham, MA, USA). Libraries, pooled according to protocol, were prepared using Illumina DNA Prep and IDT Illumina DNA/RNA UD Index Kit (Illumina, San Diego, CA, USA) and sequenced on an Illumina MiSeq. 2 × 200 platform. Raw reads underwent quality assessment with FastQC v. 0.12.1. Acceptance criteria included an average Phred quality score ≥ 30, per‐base quality not dropping below Q20 in most of the read length, and absence of adapter contamination or overrepresented sequences. Reads not meeting these thresholds were trimmed or discarded. Reads were mapped to a reference sequence (EPI_ISL412866 and EPI_ISL1653999 for RSV‐A and ‐B, respectively) using the Geneious Prime software v. 11.1 [21] to obtain consensus sequences.

### 2.4 Phylogenetic Data Sets and Cluster Analysis

RSV genotypes and clades were identified using Nextclade v2.14.1 classification [22, 23].

Multiple sequence alignment was made using MAFFT [24, 25], and the alignment was manually edited by the BioEdit v. 7.2.6.1 program [26]. Distinct data sets were constructed for each subtype.

Phylogenetic trees were constructed using IQ‐TREE v2.2.1 [27] with 1000 bootstrap replicates and visualized using FigTree (v1.4.4). GTR + F + R6 and GTR + F + I + I + R3 models were selected by ModelFinder for RSV‐A and B, respectively. International sequences of whole genomes carrying the same genotype of Italian strains obtained from Gisaid database (last accessed December 2023) were included to obtain a final data set of 841 (Italy *n* = 89, Europe *n* = 300, other continents *n* = 452; collected from February 2012 to August 2023) and 775 (Italy *n* = 136, Europe *n* = 295, other continents *n* = 344; collected from July 2013 to July 2023) sequences for RSV‐A and RSV‐B, respectively. Italian sequences included new samples collected in the 2022–2023 season (*n* = 100), previously published sequences from 2021 to 2022 season (*n* = 88) [18] and addition strains retrieved from Gisaid collected in the 2022–2023 season (*n* = 37). International strains were selected among those available to have maximum 10 sequences/year for European countries or continents.

Statistically supported clusters, including more than three sequences, were identified by using the Cluster Pickers v.1.2.3 software using 90% bootstrap support and a mean genetic distance of 1% as thresholds. Only clusters including at least one Italian sequence were selected and classified as mixed (M), containing both Italian and non‐Italian isolates in different proportions, pure Italian (IT), including only Italian genomes, or singleton (S), containing only a single Italian genome interspersed within non‐Italian sequences.

### 2.5 Phylodynamic Analysis

The cluster subsets, encompassing sequences in clusters containing at least one Italian and more than 10 strains, were used for the tMRCA (time of the most recent common ancestor) estimation, phylodynamic and phylogeographic analyses. Bayesian analyses were performed by BEAST v. 2.7.4 with the same substitution model employed for the previously described analyses. BEAST is a software widely used in molecular phylogenetics for estimating rooted, time‐calibrated phylogenies. It estimates rooted, time‐measured phylogenies using strict or relaxed molecular clock models. Beyond reconstructing phylogenies, BEAST 2 also provides a flexible framework for testing evolutionary hypotheses without assuming a single tree topology. The program employs Markov chain Monte Carlo (MCMC) methods to integrate over tree space, weighing each tree according to its posterior probability. For this study, MCMC analyses were run for 30 million generations and sampled every 3000 selecting Bayesian skyline plot (BSP) model under relaxed molecular clock. Convergence was assessed by estimating the effective sampling size (ESS) after applying a 10% burn‐in through Tracer v.1.7 software, accepting ESS of at least 200. The uncertainty of estimates was indicated with 95% highest prior density (HPD) intervals.

The birth‐death skyline model was used to estimate key epidemiological indicators, including the subtype specific reproduction number (*R*e), representing its transmissibility potential, and other epidemiological parameters such as the death/recovery rate (*δ*), the transmission rate (*λ*), the origin of the epidemic, informing on the time of initial (potentially unobserved) subtype emergence, and the sampling proportion (*ρ*), which provides insights into the overall number of individuals infected by the different subtypes during the study period. Given that the samples were collected during a short period of time, a “birth‐death skyline serial” model was used.

For the birth‐death analysis, one and two intervals and a lognormal before Re, with a mean (*M*) of 0.0 and a variance (*S*) of 1.0 were chosen, which allows the Re values to change between less than 1 and more than 7.

A lognormal prior with *M* = 4.29 and *S* = 1.0 (95% CI: 14.3–378) was used for the rate of becoming uninfectious. These values are expressed as units per year and reflect the inverse of the time of infectiousness (5.3–19 days; mean, 7.5). Sampling probability (*ρ*) was estimated assuming a prior *β* (*α* = 1.0 and *β* = 1000), estimated based on available genomes in the analyses (normalizing to 1).

### 2.6 Statistical Analysis

Descriptive analyses of demographic and clinical data are presented as median and interquartile range (IQR) when continuous and as frequency and proportion (%) when categorical. Parametric tests (*t*‐test and analysis of variance), nonparametric tests (Mann–Whitney and Kruskal–Wallis), and the Pearson *χ* ^2^ test (or Fisher exact test, when necessary) were used to compare normally distributed, non‐normally distributed continuous, and categorical variables of patients, respectively. Significance was established at a value of *p* < 0.05. Data analysis was performed using IBM SPSS Statistics version 25.

## 3 Results

### 3.1 Patient Characteristics

**Figure 1:**
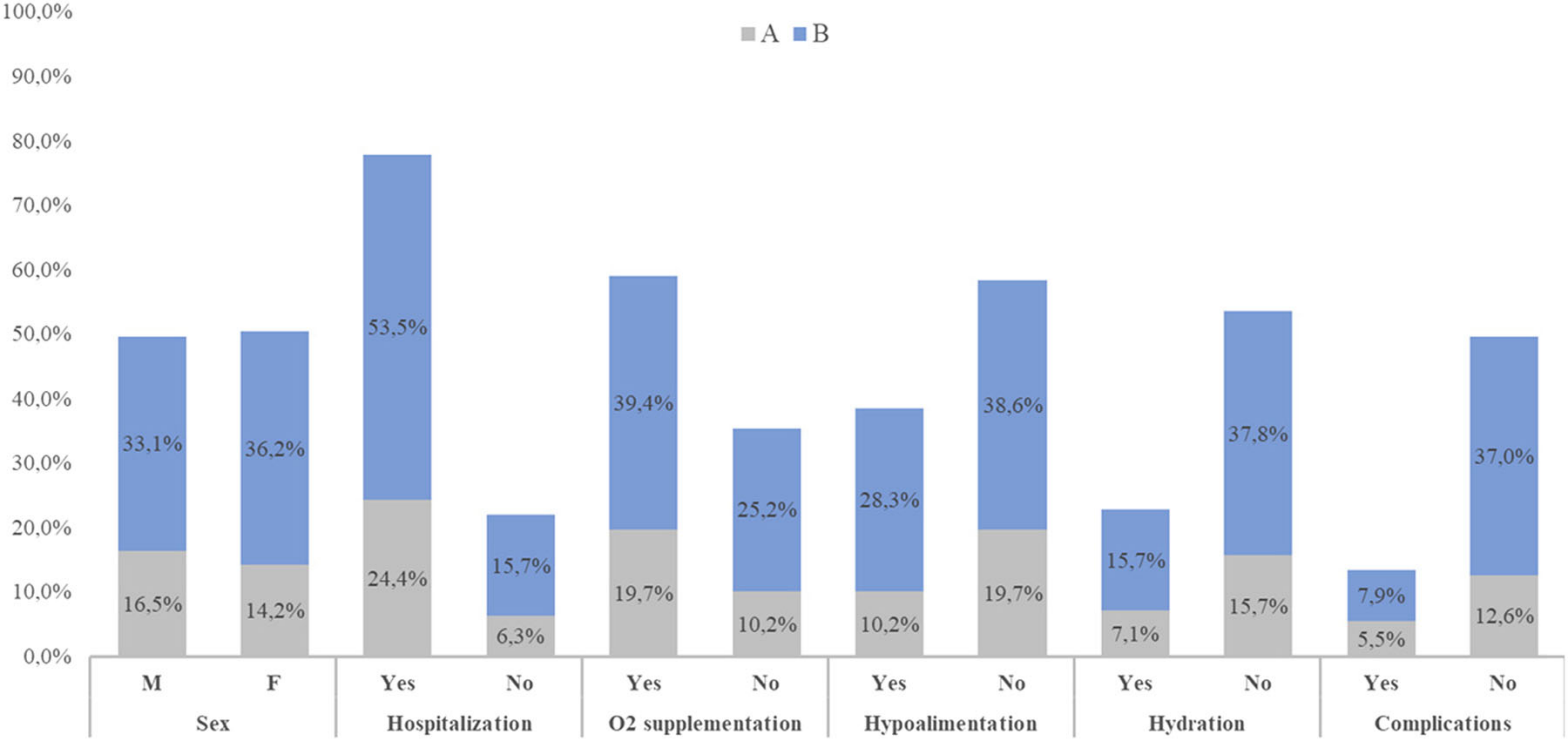
Characteristics of studied subjects stratified according to subtype.

Figure 1 shows the main characteristics of the enrolled subjects. Sexes showed an identical distribution, and the median age was 92 days (IQR: 55–202 days) without differences between females and males (96, IQR: 55–207 vs. 86, IQR: 56–186 days, respectively). Median RSV RT‐PCR cycle threshold (*C_t_*) on nasopharyngeal swabs at diagnosis was 19.6 (IQR: 18–22).

A large proportion of the subjects had required hospitalization (*n* = 99, 78%) and this proportion was twice as high in children under 3 months of age compared to those over 3 months (*n* = 69, 69.7% vs. *n* = 30, 30.3%; *p* < 0*.*0001). Indeed, age of hospitalized subjects was significantly lower than that of nonhospitalized subjects (69, IQR: 33–116 vs. 129, IQR: 67–226 days; *p* < 0*.*0001).

Among hospitalized subjects, we observed a large proportion of subjects requiring oxygen supplementation (82.8%, *n* = 82) and intravenous hydration (70.1%, 68/97). Oxygen supplementation included simply oxygen (*n* = 43), high‐flow nasal cannula (HFNC, *n* = 12), simply oxygen + HFNC (*n* = 11), continuous positive airway pressure (CPAP, *n* = 5), nasal cannula (*n* = 3), and noninvasive ventilation (NIV, *n* = 1). Even oxygen supplementation requirement was significantly higher in subjects younger than 3 months with respect to those older (64.6%, *n* = 53 vs. 35.4%, *n* = 29; *p* = 0.016). Clinical complications were observed in 21.2% (17/80) of hospitalized subjects showing a significantly higher median age compared to those without clinical complications (129, IQR: 67–226 vs. 59.5, IQR: 33–116; *p* = 0.005). Notably, 10 subjects presented acute respiratory failure and/or bronchopneumonia. Other complications included Norovirus infection (*n* = 2), acute otitis media (*n* = 2), *H. Influenzae* bacteriemia (*n* = 1), laryngitis (*n* = 1) or conjunctivitis (*n* = 1). Median duration of hospital stays and hydration was 6 days (IQR: 5–9) and 3 days (IQR: 2–4), respectively.

For those with available information, 93 were born after a full‐term pregnancy while two subjects were born at week 36; 47 had brothers or sisters while 20 were only children. Only one subject was treated with Palivizumab.

### 3.2 RT‐PCR Subtype Analysis and Whole Genome Characterization

RSV‐B subtype was more prevalent than RSV‐A (69.3%, *n* = 88 vs. 30.7%, *n* = 39). Significant lower median values of Ct were observed in subtype A compared to B at diagnosis (18.3, IQR: 17–20 vs. 19.9, IQR: 19–22; *p* = 0.010), no other significant correlations were observed between *C_t_* and clinical characteristics.

However, we did not find any significant differences in age groups and clinical characteristics between RSV‐A and RSV‐B positive patients (Figure 1).

Whole genome was obtained from 102 samples (Supporting Information: Figure S1): 31 RSV‐A (representing 79.5% of all the RSV‐A) and 71 RSV‐B (representing 80.7% of all RSV‐B positive samples). Subtype A samples belonged to two different genotypes: 29 (93.5%) were assigned to GA2.3.5 genotype (ON1 strains), and two (6%) to GA2.3.3 genotype. GA2.3.5 genotype samples were furtherly subdivided into nine different clades: A.D.3/A.D.3.1/A.D.3.3 representing one‐third of the samples (37.9%, *n* = 11) followed by A.D.5/A.D.5.2/A.D.5.3 (*n* = 8, 27.6%), A.D.1/A.D.1.7 (*n* = 7, 24.1%), and A.D.2.1 (*n* = 3, 10.3%).

On the contrary, all 71 subtype B samples were assigned to GB5.0.5a genotype (BA‐10 strain). Four clades were represented: most strains belonged to B.D.E.1 clade (84.5%, *n*= 60) while the remaining were classified as B.D.4.1/B.D.4.1.1 (*n* = 10, 14.1%), and B.D.E.3 (*n* = 1, 1.4%). No differences were observed in clinical and/or epidemiological characteristics of subjects stratifying for genotype or clade.

### 3.3 Phylogenetic Analysis of the International Data Set

Phylogenetic analysis of the RSV‐A international data set identified a total of 61 clades (with more than three strains). Italian strains (*n* = 89) were included in a total of 14 clades, largely corresponding to the whole genome clades previously described. In particular, the highest number of Italian isolates (*n* = 32, 36%), were included into the largest clade [28] corresponding to the clade A.D.5.2, followed by clusters #56 and #57 corresponding to clade A.D.3 including 14 strains (15.7%) and cluster #40 (clade A.D.1), encompassing 8 Italian sequences (9%). One cluster [29], including only three Italian strains sampled in December 2022, corresponded to the clade A.D.2.1 (Table 1, Panel A). While five clusters included sequences sampled in a single season (one in 2021–2022 and four in seasons 2022–2023), all the other clusters included isolates sampled in both seasons.

**Table 1:** Composition of clusters in terms of Italian strains involved in RSV‐A (A) and RSV‐B (B) data set, respectively.

A					
Cluster	Whole‐genome clade	IT sequences	% Italian (*n* = 89)	Season of Italians	Cluster type
#1	A.D.2.1	3	3.4	2022–2023	IT
#33	A.D	2	2.2	2022–2023	M
#40	A.D.1	4	4.5	2022–2023	M
#41	A.D.1	1	1.2	2022–2023	S
#48	A.D.1/.7	3	3.4	2022–2023	M
#51	A.D.3.3	2	2.2	2021–2022/2022–2023	M
#54	A.D.3.1	2	2.2	2022–2023	M
#56	A.D.3	9	10.1	2021–2022/2022–2023	M
#57	A.D.3	5	5.6	2021–2022/2022–2023	M
#50	A.D.3	2	2.2	2022–2023	M
#61	A.D.5.(3)	9	10.1	2021–2022/2022–2023	M
#71	A.D.5.1	5	5.6	2021–2022/2022–2023	M
#72	A.D.5.2	32	36.0	2021–2022/2022–2023	M
#73	A.D.5	8	9.0	2021–2022	M

B					
Cluster	Whole‐genome clade	IT sequences	% Italian (*n* = 136)	Season of Italians	Cluster type
#6	B.D.4.1.1	7	5.3	2021–2022/2022–2023	M
#10	B.D.4.1.1	2	1.5	2022–2023	M
#14	B.D.4.1.1	2	1.5	2021–2022	M
#23	B.D.4.1.1	23	17.4	2021–2022/2022–2023	M
#30	B.D.4.1.1	4	3.0	2022–2023	M
#33	B.D.4.1	4	3.0	2021–2022/2022–2023	M
#35	B.D.E.1	5	3.8	2022–2023	M
#38	B.D.E.1	5	3.8	2021–2022/2022–2023	M
#41	B.D.E.1	23	17.4	2021–2022/2022–2023	M
#43	B.D.E.1	1	0.8	2022–2023	S
#44	B.D.E.1	18	13.6	2021–2022/2022–2023	M
#46	B.D.E.1	1	0.8	2022–2023	S
#47	B.D.E.1	19	14.4	2021–2022/2022–2023	M
#50	B.D.E.1	14	10.6	2021–2022/2022–2023	M
#51	B.D.E.1	3	2.3	2022–2023	M
#52	B.D.E.1	1	0.8	2022–2023	S

Abbreviations: IT, pure Italian clusters; M, mixed clusters; S, singleton, containing only one Italian isolate

RSV‐B international data set showed 42 significant clusters (with more than three sequences). Italian strains (*n* = 136) were included in 16 mixed clusters, containing both Italian and European isolates. Largest number of Italian strains were included in clusters #23, corresponding to B.D.4.1.1 and in cluster #41, corresponding to B.D.E.1, including 23 genomes each (16.9% of the Italian sequences), followed by clusters #47 (*n* = 19), #44 (*n* = 18) and #50 (*n* = 15), all corresponding to clade B.D.E.1, and including respectively 14%, 13.2% and 11% of the Italian patients. All main clusters contained isolates of both seasons, except for one [25] which included only season 2022–2023 isolates (Table 1, Panel B).

### 3.4 Bayesian Phylogenesis of the Clusters Including Italian Genomes

**Figure 2:**
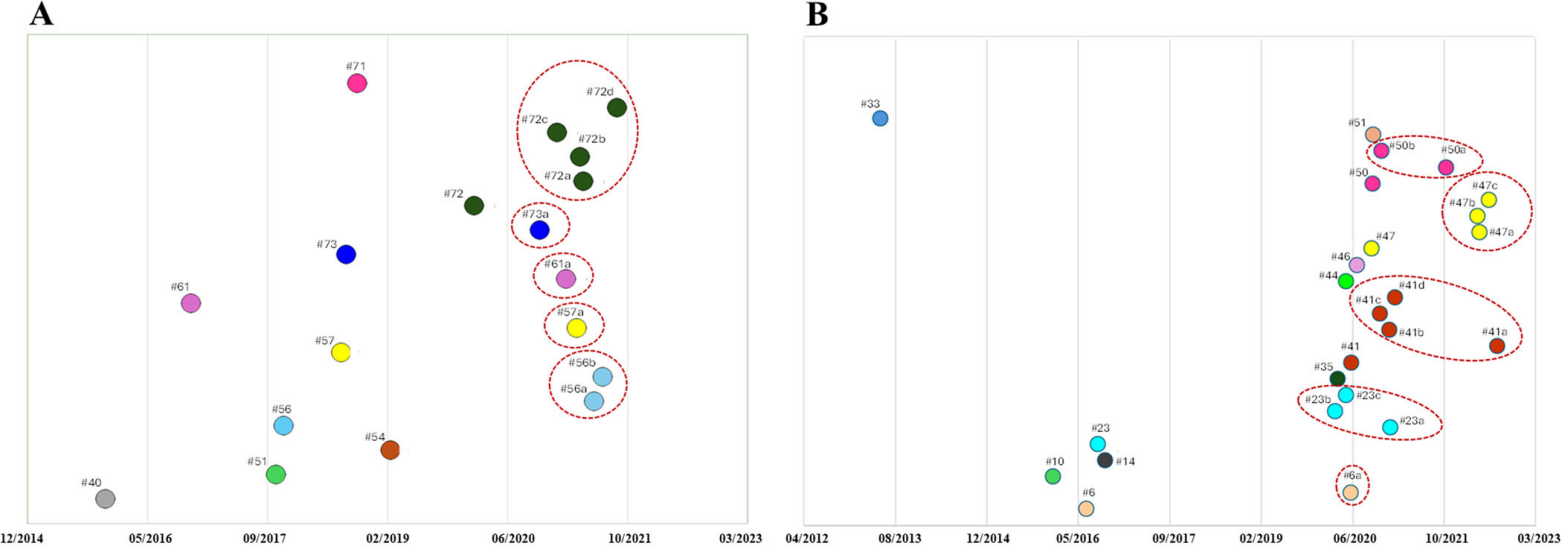
Graphical representation of mean tMRCA estimation of the main clusters and subclades of RSV‐A (A) and RSV‐B (B) data set. The dotted red lines indicate the subclades.

The Bayesian analysis of the RSV‐A clusters estimated an evolutionary rate of 1.13 × 10^−3^ sub/site/year (95% HPD: 9.8726 10^–4^ to 1.2777 × 10^–3^), resulting in a tree‐root tMRCA corresponding to November 2011 (95% HPD: February 2009–May 2014). The tMRCA of the RSV‐A clusters dated between November 2015 [30], 95% HPD: April 2015‐ October 2016] and January 2020 [28], 95% HPD: May 2019–September 2020]. The duration of the clusters ranged between 3 and 7 years; the longer‐lasting clusters were #40 (7 years) and #61 (6 years). Moreover, Italian strains within the single clusters tended to group together forming monophyletic groups including only Italian strains. These groups (called subclades) were nine in RSV‐A, including from two to nine Italian isolates, collected during the 2021–2022 season in six cases, or during the 2022‐2023 season in one case and in both seasons in two cases. The tMRCAs of these groups varied from October 2020 to September 2021 (median April 2021) independently from the sampling season (Table 2, Panel A; Figure 2A).

**Table 2:** tMRCA estimation of the main clusters of RSV‐A (A) and RSV‐B (B) data set with the relative 95% HPD and duration in the time.

A							
Cluster	Node	tMRCA	95% HPD	No. of total sequences	No. of IT sequences	pp	Years
#40	**Root**	17/11/2015	04/04/2015–10/06/2016	63	4	1	7
**#51**	Root	27/10/2017	04/05/2017–20/03/2018	36	2	0.7	5
**#54**	Root	16/02/2019	31/08/2018–11/07/2019	20	2	1	4
**#56**	**Root**	**28/11/2017**	**23/05/2017–02/05/2018**	**35**	**9**	**0.9**	**5**
**pure IT**	a	13/06/2021	20/11/2020–30/12/2021	/	4	1	/
	b	20/07/2021	22/04/2021–06/10/2021	/	3	1	/
**#57**	**Root**	**26/07/2018**	**11/09/2017‐14/07/2019**	**11**	**5**	**1**	**4**
**pure IT**	a	03/04/2021	06/11/2020–03/09/2021	/	3	1	/
**#61**	**Root**	**07/11/2016**	**07/09/2015–03/05/2019**	**39**	**9**	**1**	**6**
**pure IT**	b	17/02/2021	27/07/2020–25/08/2021	/	7	1	/
**#71**	Root	01/10/2018	19/02/2018–03/05/2019	19	5	1	4
**#72**	**Root**	**31/01/2020**	**31/05/2019–21/09/2020**	**85**	**32**	**1**	**3**
**pure IT**	a*	30/04/2021	02/02/2021–23/07/2021	/	5	1	/
	b*	16/04/2021	17/01/2021–14/07/2021	/	7	1	/
	c	11/01/2021	17/09/2020–31/03/2021	/	9	0.8	/
	d	17/09/2021	05/07/2021–07/11/2021	/	3	1	/
**#73**	**Root**	**16/08/2018**	**20/01/2018–09/03/2019**	**12**	**8**	**1**	**3**
**pure IT**	c	30/10/2020	11/03/2020–09/05/2021	/	5	1	/

B							
Cluster	Node	tMRCA	95% HPD	No. of Total sequences	No. of IT sequences	pp	Years
**#6**	**Root**	**24/06/2016**	**08/02/2016–24/12/2016**	**18**	**7**	**1**	**6**
pure IT	a	09/06/2020	29/01/2020–19/10/2020	/	6	1	/
#10	Root	25/12/2015	01/09/2015–02/05/2016	63	2	0.7	7
#14	Root	06/10/2016	30/05/2016–29/05/2017	12	2	1	6
**#23**	**Root**	**26/08/2016**	**18/04/2016–27/03/2017**	**43**	**23**	**1**	**6**
pure IT	a	11/01/2021	26/09/2020–15/04/2021	/	15	1	/
	b	17/03/2020	07/08/2019–18/09/2020	/	4	1	/
	c	14/05/2020	06/10/2019–26/11/2020	/	3	1	/
#33	Root	25/05/2013	03/01/2013–29/09/2013	42	4	0.6	9
#35	Root	30/03/2020	07/12/2019–25/07/2020	32	5	1	3
**#41**	**Root**	**11/06/2020**	**11/03/2020–27/09/2020**	**34**	**23**	**1**	**2**
pure IT	a	20/08/2022	11/06/2022–26/10/2022	/	4	1	/
	b	07/01/2021	03/11/2020–09/03/2021	/	6	1	/
	c	16/11/2020	31/08/2020–04/01/2021	/	6	1	/
	d	07/02/2021	30/10/2020–31/05/2021	/	4	0.88	/
#44	Root	15/05/2020	10/02/2020–23/08/2020	64	18	1	3
#46	Root	14/07/2020		15	1	0.5	2
**#47**	**Root**	**01/10/2020**	**18/07/2020–15/12/2020**	**41**	**19**	**1**	**2**
pure IT	a	14/05/2022	11/01/2022–29/08/2022	/	4	0.98	/
	b	04/05/2022	22/01/2022–04/08/2022	/	6	1	/
	c	06/07/2022	28/03/2022–30/09/2022	/	4	1	/
**#50**	**Root**	**06/10/2020**	**20/07/2020–21/12/2020**	**24**	**14**	**1**	**2**
pure IT	a*	12/11/2021	18/06/2021–13/03/2022	/	7	1	/
	b	25/11/2020	20/07/2020–20/12/2020	/	4	1	/
#51	Root	11/10/2020	27/07/2020–28/12/2020	21	3	1	2

Abbreviations: HPD, highest posterior density; pp, posterior probability.

The analysis of the RSV‐B clusters showed evolutionary rate estimates of 1.2 × 10^−3^ sub/site/year (95% HPD: 1.0923–1.2895 × 10^−3^) resulting in a mean tMRCA of the tree root going back in 2012 (95% HPD: August 2011–September 2013) and of the single clusters varying from May 2013 [31], 95% HPD: January–September 2013] to July 2020 [25], #50, #51, 95% HPD: July–December 2020]. The clusters persistence was around 2‐9 years, and the most enduring clusters were #33 (9 years) and #10 (7 years).

Even in the case of RSV‐B phylogenesis, some of the Italian isolates tended to group together, forming 13 Italian monophyletic subclades including from 3 to 15 genomes per group collected only during the 2021–2022 season in four cases, during the 2022–2023 season in seven cases and during both seasons in two cases. The tMRCAs of these groups ranged from as early as 2019 (May) to beyond 2022 (August), but while the estimated median tMRCAs of the groups that included strains obtained in the 2021–2022 season or in both seasons fell around mid‐2020, that of the groups including samples obtained during the most recent 2022–2023 season dated around mid‐2021 (Table 2, Panel B; Figure 2B).

**Figure 3:**
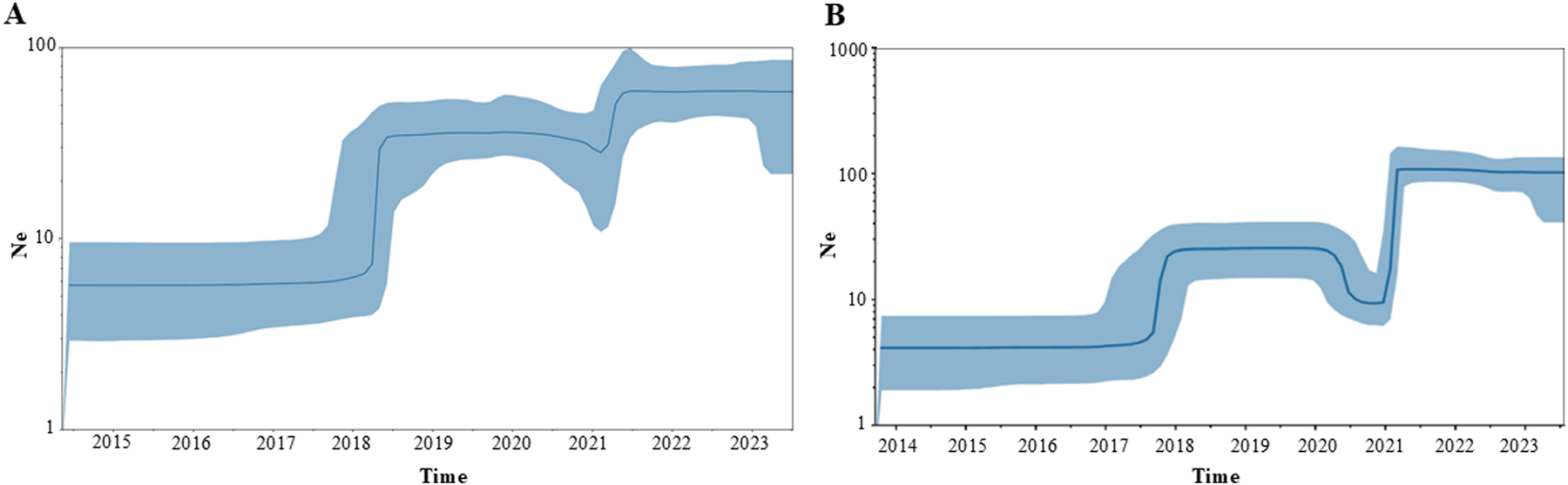
Bayesian Skyline plot of RSV‐A (A) and RSV‐B (B). The *Y*‐axis indicates effective population size (N_e_) and the *X‐*axis shows the time in fraction of years. The thick solid line represents the median value of the estimates, and the gray area the 95% HPD.

Phylodynamic analysis by coalescent skyline indicated for both subtypes, two phases of exponential growth in the number of infections in early 2018 and 2021, followed by stationary phases. In both cases, a brief period of reduction in the estimated effective number of cases (bottleneck) was evident in the years 2020 and 2021 (highlighted in Figure 3).

### 3.5 Estimates of the Effective Reproductive Number for RSV‐A and RSV‐B Italian Data Set

To estimate the population dynamics of the epidemics in Italy, we analyzed the Italian subsets including 89 RSV‐A genomes and 136 RSV‐B genomes collected in 2021–2022 and 2022–2023, by a Bayesian birth‐death skyline model.

**Figure 4:**
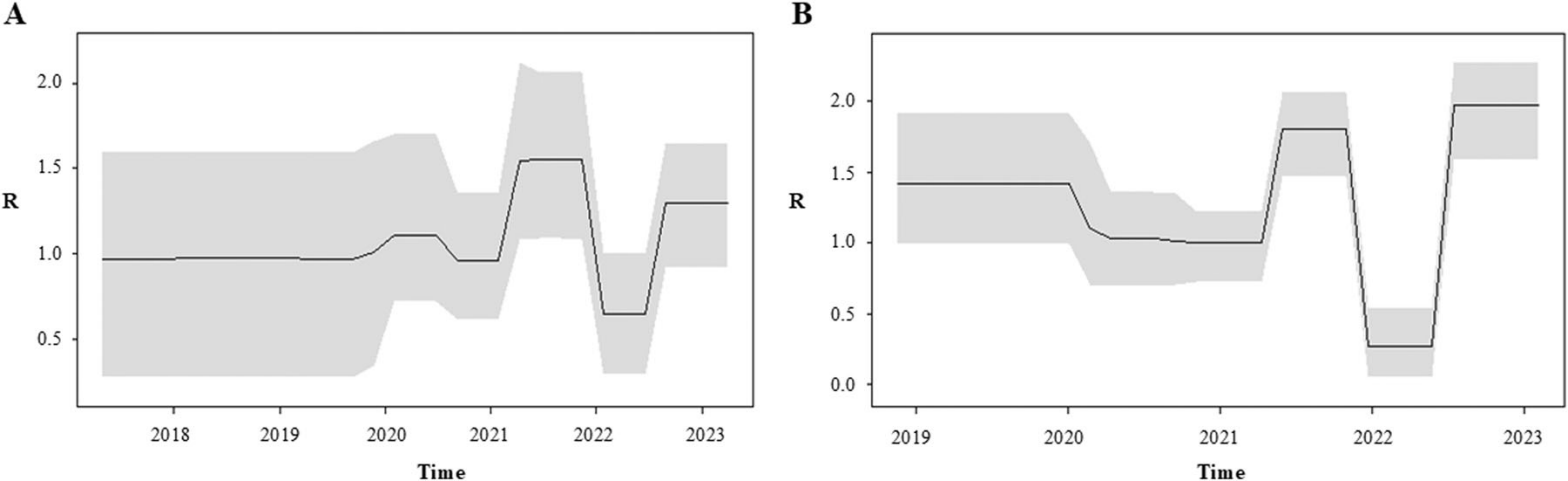
Birth‐death skyline plot of RSV‐A (A) and RSV‐B (B), in relation to time (*x*‐axis) and the effective reproduction rate (*R_e_*) (*y*‐axis).

The curves representing the dynamic of the effective reproduction number (*R*_e_) are reported in the Figure 4.

The effective reproduction number of RSV‐A reached a value of 1.39 (95% HPD: 0.8–2.1) in 2021, then decreased < 1 and grew again between 2022 and 2023 reaching the value of 1.2 (95% HPD: 0.9–1.6) (Figure 4A). On the contrary, RSV‐B showed a multiphasic trend with a value > 1 already in the years preceding the pandemic (mean *R_e_* estimate = 1.43; 95% HPD: 0.99–1.9), followed by a decrease during 2020‐2021 period and an increase starting in the second half of 2021 (mean *R*_e_ estimate = 1.79; 95% HPD: 1.48–2.07). Then, a further decrease in the first half of 2022, and a renewed (and seemingly more pronounced) growth between the second half of 2022 and the beginning of 2023 (*R*_e_ = 1.96; 95% HPD: 1.58–2.27) (Figure 4B).

### 3.6 Mutations and Variability Analysis

A comparison of the predicted amino acid sequences of the RSV‐A (GA2.3.5 genotype) and RSV‐B (GB5.0.5.a genotype) isolates to those of reference sequences revealed 78 and 31 mutation along genome (Table 3, Parts A and B) with a percentage ≥ 10% in the 2022–2023 Italian strains. Around half of the mutations were observed in the G gene considering both subtypes (*n* = 36, 46.2% and *n* = 16, 51.6% for RSV‐A and RSV‐B, respectively).

**Table 3:** Amino acid substitutions found with a frequency of ≥ 10% along genome in RSV‐A (A) and RSV‐B (B) Italian sequences stratified according to season.

A				
Gene	Mutation	Total *n* = 89	Season 2021–2022 *n* = 49	Season 2022–2023 *n* = 40
		% (*n*)	% (*n*)	% (*n*)
**NS‐1**	V82I	5.6 (5)	10.2 (5)	0 (0)
**N**	V352A	100 (89)	100 (49)	100 (40)
**P**	L55P	98.9 (88)	100 (49)	97.5 (39)
	T69A	4.5 (4)	0 (0)	10 (4)
	T69I	36 (32)	53.1 (26)	15 (6)
	T72A	4.5 (4)	0 (0)	10 (4)
	T92M	18 (16)	16.3 (8)	20 (8)
**M**	M43I	18 (16)	2 (1)	3.7 (15)
	M73L	50.6 (45)	77.5 (38)	17.5 (7)
	E178G	4.5 (4)	0 (0)	10 (4)
	M254I	9 (8)	2 (1)	17.5 (7)
**SH**	I38S	4.5 (4)	0 (0)	10 (4)
**G**	A57V	50.6 (45)	77.6 (38)	17.5 (7)
	A65T	7 (6)	0 (0)	15 (6)
	H67N	4.5 (4)	0 (0)	10 (4)
	P71L	100 (89)	100 (49)	100 (40)
	H90Y	98.9 (88)	98 (48)	100 (40)
	L101F	96.6 (86)	98 (48)	95 (38)
	T113I	21.3 (19)	16.3 (8)	27.5 (11)
	V131D	21.3 (19)	16.3 (8)	27.5 (11)
	I134K	94.4 (84)	98 (48)	90 (36)
	L142S	18 (16)	6.1 (3)	32.5 (13)
	N178G	21.3 (19)	16.3 (8)	27.5 (11)
	P206Q	60.7 (54)	79.6 (39)	37.5 (15)
	T207N	6.7 (6)	12.2 (6)	0 (0)
	K209R	50.6 (45)	77.5 (38)	17.5 (7)
	P222L	4.5 (4)	0 (0)	10 (4)
	G224E	94.4 (84)	98 (48)	90 (36)
	P230T	5.6 (5)	0 (0)	10 (4)
	S243I	98.9 (88)	98 (48)	100 (40)
	R244K	8 (7)	2 (1)	15 (6)
	L248I	60.7 (54)	80 (39)	37.5 (15)
	H258Q	21.3 (19)	16.3 (8)	27.5 (11)
	K262E	92.1 (82)	96 (47)	87.5 (35)
	I265L	97.8 (87)	98 (48)	97.5 (39)
	H266L	21.3 (19)	16.3 (8)	27.5 (11)
	Y273H	13.5 (12)	18.3 (9)	7.5 (3)
	P274L	24.7 (22)	16.3 (8)	35 (14)
	P276Q	9 (8)	0 (0)	20 (8)
	V279A	50.6 (45)	80 (39)	15 (6)
	Y280H	62.9 (56)	80 (39)	42.5 (17)
	D284G	11.2 (10)	0 (0)	25 (10)
	V303A	8.9 (8)	0 (0)	20 (8)
	Y304H	12.4 (11)	0 (0)	27.5 (11)
	L310P	64 (47)	80 (39)	45 (18)
	S311P	11.2 (10)	2 (1)	22.5 (9)
	L314P	16.9 (15)	4.1 (2)	32.5 (13)
	T319I	50.6 (45)	77.6 (38)	17.5 (7)
	T319S	4.5 (4)	0 (0)	10 (4)
	T320A	73 (65)	82 (40)	62.5 (25)
**F**	P4S	8 (7)	2 (1)	15 (6)
	T12I	21.3 (19)	16.3 (8)	27.5 (11)
	L15F	10.1 (9)	2 (1)	20 (8)
	A103T	36 (32)	53.1 (26)	15 (6)
	M115T	6.7 (6)	12.3 (6)	0 (0)
	T122A	60 (53)	80 (39)	35 (14)
**M2‐1**	K52R	10.1 (9)	2 (1)	20 (8)
	N117K	5.6 (5)	10.2 (5)	0 (0)
	S176P	98 (87)	98 (48)	97.5 (39)
**M2‐2**	N18S	8 (7)	2 (1)	15 (6)
	Y26C	63 (56)	45 (22)	85 (34)
	S46N	92.1 (82)	98 (48)	85 (34)
	H47R	4.5 (4)	0 (0)	10 (4)
	D64G	4.5 (4)	0 (0)	10 (4)
	T79A	19.1 (17)	16.3 (8)	22.5 (9)
	T79N	4.5 (4)	0 (0)	10 (4)
	I7V	6.7 (6)	12.3 (6)	0 (0)
**L**	E81D	6.7 (6)	2 (1)	12.5 (5)
	N146D	10.1 (9)	0 (0)	22.5 (9)
	P174L	98.8 (88)	98 (48)	100 (40)
	T182A	10.1 (9)	2 (1)	20 (8)
	T182S	10.1 (9)	0 (0)	22.5 (9)
	R259K	97.7 (87)	98 (48)	97.5 (39)
	K376R	6.7 (6)	0 (0)	15 (6)
	Y601H	92.1 (82)	96 (47)	87.5 (35)
	I760V	8 (7)	2 (1)	15 (6)
	L838M	22.5 (20)	18.4 (9)	27.5 (11)
	K1028R	8 (7)	14.3 (7)	0 (0)
	L1441Q	96.6 (86)	96 (47)	97.5 (39)
	I1656V	10.1 (9)	0 (0)	22.5 (9)
	K1664N	10.1 (9)	0 (0)	22.5 (9)
	N1726G	85.4 (52)	73.5 (36)	40 (16)
	N1726S	38.2 (34)	20.4 (10)	60 (24)
	E1728G	71 (63)	63.3 (31)	80 (32)
	G1734D	96.6 (86)	94 (46)	100 (40)
	V1946D	56.2 (50)	73.5 (36)	40 (14)

B				
Gene	Mutation	Total *n* = 136	Season 2021–2022 *n* = 39	Season 2022–2023 *n* = 97
		% (*n*)	% (*n*)	% (*n*)
**NS‐2**	M1V	5.1 (7)	15.4 (6)	1 (1)
	N5K	16.9 (23)	28.2 (11)	12.4 (12)
**N**	V90A	4.4 (6)	15.4 (6)	0 (0)
	V97I	99.3 (135)	97.4 (38)	100 (97)
**M**	T89I	6.7 (9)	12.8 (5)	4.1 (4)
**G**	A74V	99.3 (135)	97.4 (38)	100 (97)
	*S100G	59.6 (81)	48.7 (19)	63.9 (62)
	Q104H	4.4 (6)	12.8 (5)	1 (1)
	H128Y	5.1 (7)	12.8 (5)	2.1 (2)
	T131A	73.5 (100)	56.4 (22)	80.4 (78)
	G135S	4.4 (6)	12.8 (5)	1 (1)
	I137T	73.5 (100)	56.4 (22)	80.4 (78)
	T141K	4.4 (6)	12.8 (5)	1 (1)
	*N143S	11 (15)	0 (0)	15.5 (15)
	T198A	2.9 (4)	10.3 (4)	0 (0)
	P214S	66.9 (91)	48.7 (19)	74.2 (72)
	*L217P	23 (31)	35.9 (14)	17.5 (17)
	P221L	67.6 (92)	48.7 (19)	75.3 (73)
	N228K	10.3 (14)	0 (0)	14.4 (14)
	P235L	4.4 (6)	12.8 (5)	1 (1)
	D251N	6.6 (9)	12.8 (5)	4.1 (4)
	I252T	72.1 (98)	59 (23)	77.3 (75)
	K256N	61.8 (84)	46.2 (18)	68 (66)
	S265P	15.4 (21)	12.8 (5)	16.5 (16)
	I268T	80.1 (109)	56.4 (22)	89.7 (87)
	A269V	13.2 (18)	0 (0)	18.6 (18)
	S275P	64 (87)	46.2 (18)	71.1 (69)
	Y285H	64.7 (88)	48.7 (19)	71.1 (69)
**F**	A19V	4.4 (6)	12.8 (5)	1 (1)
	R42K	12.5 (17)	5.1 (2)	15.5 (15)
	A111V	11.8 (16)	0 (0)	16.5 (16)
	S190N	71.3 (97)	51.3 (20)	79.4 (77)
	R209Q	5.9 (8)	12.8 (5)	3.1 (3)
	S211N	72.1 (98)	51.3 (20)	80.4 (78)
	S389P	68.4 (93)	51.3 (20)	75.3 (73)
	M526V	4.4 (6)	12.8 (5)	1 (1)
**M2‐2**	I2T	97.8 (133)	94.9 (37)	99 (96)
	M27T	65.4 (89)	51.3 (20)	71.1 (69)
	D35N	67.6 (92)	51.3 (20)	74.2 (72)
	C49F	67.6 (92)	51.3 (20)	74.2 (72)
**L**	V164M	3.7 (5)	10.3 (4)	1 (1)
	K570R	71.3 (97)	51.3 (20)	79.4 (77)
	A675V	4.4 (6)	15.4 (6)	0 (0)
	I677V	2.9 (4)	10.3 (4)	0 (0)
	T1596I	2.9 (4)	10.3 (4)	0 (0)
	V1716I	4.4 (6)	12.8 (5)	1 (1)
	R1759K	69.1 (94)	43.6 (17)	79.4 (77)
	V1965A	4.4 (6)	12.8 (5)	1 (1)
	V1965I	13.2 (18)	5.1 (2)	16.5 (16)
	T1987I	77.2 (105)	64.1 (25)	82.5 (80)

*Note:* In pink are highlighted mutations found only in the 2021–2022 season, in light blue those present only in the 2022–2023 season, in gray mutations that increased their proportion in the last season, in green those that decrease their proportion in the 2022–2023 season. Mutations largely present in both seasons are reported in red.

Comparing isolates of the seasons 2021–2022 and 2022–2023, season 22‐23 sequences in A subtype had a greater number of mutations (*n* = 78) comparing to seasons 2021–2022 (*n *= 52) while in the RSV‐B the largest number of mutations was observed in the 2021–2022 samples (*n* = 43). In RSV‐A, 21 mutations newly emerged in isolates of 2022–2023 season while 6 mutations were distinctive of the 2021–2022 season and then disappeared. Some mutations increased (*n* = 26, range: 1.7%–40%) or decreased (*n* = 16, range: 10.8%–65%) their proportion between two seasons and 17 were largely present in quite all Italians. None of the observed substitutions were associated with resistance to the monoclonal antibodies in use. In subtype B, three mutations characterized 2022‐2023 season isolates, while six mutations were observed only in the 2021–2022 season. Some mutations increased (*n* = 22, range: 3.7%–35.8%) or decreased (*n* = 15, range: 8.7%–18.4%) their proportion between two seasons and three were largely present in quite all Italians. One mutation in nirsevimab‐targeted site (S211N, in protein F) was observed with a proportion of 53.8% and 80% in 2021–2022 and 2022–2023 isolates, respectively.

The analysis of the selective pressure showed a total of three sites under significant positive selective pressure, all located in the G protein (S100G, N143S, and L217P), in RSV‐B Italian data set.

## 4 Discussion

The present study considered a large series of children afferent to a pediatric tertiary center, which represents one of the most important clinical centers for the hospitalization and care of children in the city of Milan and Lombardy. The results confirmed previous data [29] indicating that hospitalization due to RSV infection was typically seen in children under 1 year of age, with more than half of the cases observed in children under 3 months of age. However, we did not find an association between subtype and disease severity or morbidities such as prematurity as observed in other works [32].

While in the 2021–2022 season the two subtypes A and B were equally prevalent in our case series and in other Italian reports [18, 33], in the 2022–2023 season subtype B prevailed on subtype A. The most frequently found genotypes, GA2.3.5 and GB5.0.5a, correspond to those currently predominant globally from about 15 years [34, 35]. Two samples of subtype A belonged to genotype GA2.3.3, a genotype that was prevalent in 2009–2010 season in Italy [36], however patients with this genotype did not exhibit distinctive characteristics compared to the other subjects.

Genetic analysis suggested a significant increase in nucleotide variability in the most recent samples and especially in subtype A, which had more than twice the number of substitutions compared to subtype B. The G gene is the most variable gene, as confirmed by the large number of amino acid substitutions observed in this portion compared to the more conserved F or L proteins. In Italy, the preventive strategy with monoclonal antibody palivizumab was reserved only for infants with conditions of increased risk, while nirsevimab is recently available for prophylaxis in all neonates and infants. Since all samples analyzed in this study were collected before the introduction of nirsevimab, our cohort reflects the preprophylaxis landscape, providing a valuable baseline for future post‐prophylaxis comparisons. The mutation S211N, found in the present case file in a large proportion of subjects, is already reported in the literature as a substitution fixed in the F gene of recent RSV‐B lineages [37]. Despite the little effect of that substitution on the neutralizing potency of nirsevimab [38], the mutational pattern of RSV needs to be monitored, especially regarding the antigenic sites of the F protein, also in light of the recent global use of this antibody and vaccination.

Phylogenetic analysis showed that the Italian sequences were mainly distributed into mixed clusters, including both Italian and non‐Italian genomes, a half of which were present in both seasons, confirming that, similarly to what was already described for the 2021–2022 season [18], even the 2022–2023 RSV season was driven by multiple introductions of international clusters having tMRCA estimates dating back from 2015 to 2021 that probably continued to circulate throughout the Europe during the pandemic restriction period. Moreover, several of the Italian strains tended to group together, forming pure Italian clades within the larger international clusters, probably representing local chains of transmission. Interestingly, a few of them (three for RSV‐A and two for RSV‐B) included isolates sampled in both seasons, suggesting the existence of local strains persisting for more than one season.

To reconstruct the phylodynamic of the RSV‐A and RSV‐B in Italy we used two complementary Bayesian approaches: the coalescent and the birth‐death skyline. The first approach allowed to estimate the changes in effective number of infections and the second the changes in the effective reproductive number (*Re*). Interestingly, for both RSV‐A and RSV‐B the skyline plot showed a first peak in transmissions between 2017 and 2019 followed by a temporary reduction (a bottleneck) in 2021 coinciding with the widespread use of nonpharmaceutical control measures against COVID‐19.

In agreement with these results, the Re estimates showed fluctuating values in relation to the period intra or interepidemic. Particularly, for RSV‐A the Re value rose above 1 in the second half of 2021 (1.4, 0.8–2.1), dropped below 1 in 2022, and then climbed again (1.2, 0.9–1.6) at the end the same year and the beginning of 2023.

For RSV‐B we estimated a Re higher than 1 in 2019 until the beginning of 2020, when it decreased below 1 until the first peak in the second half of 2021, with an average value of 1.8 (1.5–2.1), and a new peak at the end of 2022 and the beginning of 2023, when it reached an even sharper peak (1.96, 1.6–2.3).

These results are in line with the observation of a change in the seasonal pattern of RSV in Italy and all Europe, with an early peak of RSV in late autumn [16]. Furthermore, the higher values of Re estimated for RSV‐B, particularly in the 2022–2023 season, is consistent with the observation that the 2022–2023 season was characterized by a predominance of RSV‐B. This was also evident from the significantly higher number of infections in the RSV‐B coalescent skyline compared to RSV‐A.

Moreover, the Re estimates agree with the lowest values obtained by several authors on the basis of epidemiological data using compartmental models [39, 40, 41]. In detail, Otomaru at al., [39] studying household cases among children, were able to observe higher average values of R_0_ in RSV‐B (1.04–1.76) compared to RSV‐A (0.92–1.33), similarly to what observed in our study. On the other hand, White has shown that the use of different compartmental models, especially whether or not they incorporate partial or waning immunity, can give very different average reproductive number estimates [42].

The main limitation of this study was that it refers to a single pediatric clinical center of North Italy and included a high prevalence of hospitalized patients, limiting our ability to study the association of subtypes and/or mutants with the severity of the disease. The included population might not be representative of the general population of RSV‐infected infants in Italy, as all included infants were recruited in a hospital (inpatient or outpatient) and not in primary health care.

Moreover, the time window of samples′ collection covers partial RSV season, however months in which swabs were collected represent the peak incidence of RSV circulation in Italy since 2021. In fact, in Italy, as in many other countries, in the post‐pandemic era, peak incidence of RSV infection has shown an anticipation respect to pre‐pandemic era.

However, the strength of our study is the analysis of a large cohort of pediatric subjects for whom clinical and genomic data were analyzed.

In conclusion, the current phylodynamic analysis suggests that the widespread circulation of RSV following the lifting of pandemic restrictions is partly due to the introduction of viral strains that have been circulating across Europe for at least 8 years, and partly to strains that exhibit a degree of persistence in our region from one season to the next. Nevertheless, the alternation of seasons with a predominance of one or the other subtype is confirmed, although the peak of the transmissions seems to be shifted to late autumn and no longer to winter, as in prepandemic times. Further studies involving larger number of patients with different level of infection severity are needed to understand whether the antigenic diversity of RSV may have an impact on the rate of hospitalization.

## Author Contributions

Alessia Lai, Annalisa Bergna, Gian Vincenzo Zuccotti, and Gianguglielmo Zehender designed research. Alessia Lai, Annalisa Bergna, Carla della Ventura, and Gianguglielmo Zehender performed research and analyzed data. Valentina Fabiano, Alice Romero, Martina Loiodice, Angela Maria Dolores Boria, Alessandro Campagnoli, Felicia Stefania Falvella, Alberto Dolci, and Gian Vincenzo Zuccotti collected samples and data. Alessia Lai, Annalisa Bergna, Gian Vincenzo Zuccotti, and Gianguglielmo Zehender wrote the original draft. All authors review, edited, and approved the final manuscript.

## Ethics Statement

The study was approved by the Ethics Committee of Lombardy region (Milan, Italy) (no. CET 135‐2023). All samples were collected with the consent of patients or legal guardians. No additional samples were required and those analyzed were obtained using a leftover of diagnostic testing stored at −80°C. Clinical information and demographic data for each patient were collected by hospital staff and physicians. The study was conducted in compliance with Good Clinical Practice and the declaration of Helsinki.

## Consent

For all participants, informed consent was obtained from the parents or legal guardians. All data used in this study were previously anonymized as required by the Italian Data Protection Code (Legislative Decree 196/2003) and the general authorizations issued by the Data Protection Authority.

## Conflicts of Interest

The authors declare no conflicts of interest.

## Supporting information


**Supporting Figure S1:** Flow diagram of analyzed samples.

**Supporting Table S1:** Primer pool design for RSV‐A amplification.

**Supporting Table S2:** Primer pool design for RSV‐B amplification.

## Data Availability

The data that support the findings of this study are available from the corresponding author upon reasonable request. Whole genome sequences were submitted to GISAID [8] with the following accession IDs: EPI_ISL_19678001‐EPI_ISL_19678071 and EPI_ISL_19677914‐EPI_ISL_19677942. Datasets and performed analyses are available upon request.
