# Supplementary figures and images for: Respiratory Syncytial Virus (RSV) in an Italian Pediatric Cohort: Genomic Analysis and Circulation Pattern in the Season 2022–2023
Source: J Med Virol. 2025 Oct 23;97(11):e70660. doi: 10.1002/jmv.70660 (PMC12548520; doi:10.1002/jmv.70660)

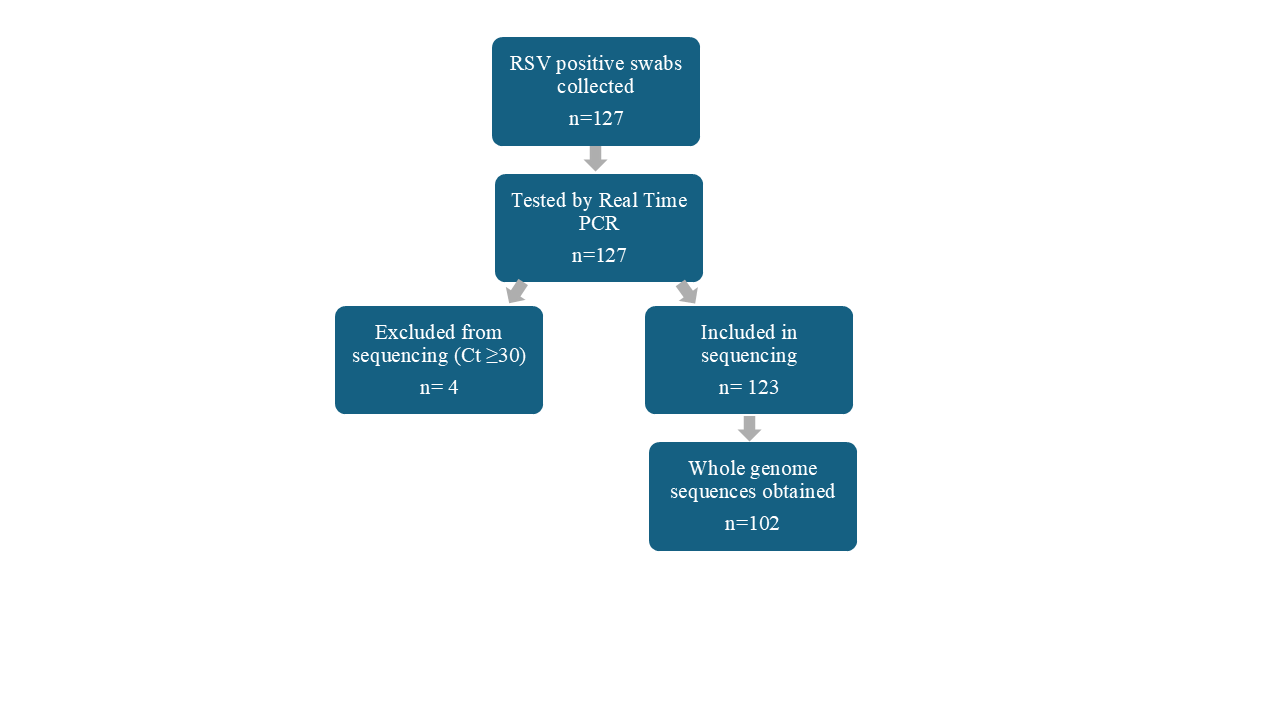

Supplement: Supplementary file 1 — Supporting Figure S1: Flow diagram of analyzed samples. [file JMV-97-e70660-s001.tif]
