# Supplementary material for: Respiratory Syncytial Virus (RSV) in an Italian Pediatric Cohort: Genomic Analysis and Circulation Pattern in the Season 2022–2023
Source: J Med Virol. 2025 Oct 23;97(11):e70660. doi: 10.1002/jmv.70660 (PMC12548520; doi:10.1002/jmv.70660)
Supplement: Supplementary file 2 — Supporting Table S1: Primer pool design for RSV‐A amplification. [file JMV-97-e70660-s002.docx]

Supplementary Table S1. Primer pool design for RSV-A amplification.

| **Pool name** | **Sequence (5'→3')** | **Genome position (nt)** | **Amplified size** |
| --- | --- | --- | --- |
| poolOne_RSVA | ACGSGAAAAAATGCGTACAAC | 1-21 | 834 |
| poolOne_RSVA | TAGTTTCATTTCATAGTTGACCAG | 811-834 |  |
| poolOne_RSVA | GTAACAACACATCGTCAAGACATTA | 1429-1453 | 965 |
| poolOne_RSVA | CTGTTGTTTGCRTCTTCTCCA | 2373-2393 |  |
| poolOne_RSVA | TGTCTCTCAATCCAACATCAGAG | 2987-3009 | 958 |
| poolOne_RSVA | GCTCCAAGATCTACTATGAATTGAC | 3920-3944 |  |
| poolOne_RSVA | AYGTATTCCATAACAAAACCTTTGAG | 4446-4471 | 902 |
| poolOne_RSVA | GTGGTTTGAGGTTTGRGATC | 5328-5347 |  |
| poolOne_RSVA | CTGTAACAGAATTGCAGTTGCT | 5927-5948 | 992 |
| poolOne_RSVA | TTTGCCATAGCATGACACAAT | 6898-6918 |  |
| poolOne_RSVA | TCAACTGAGTGGTATAAATAATATTGC | 7347-7373 | 1004 |
| poolOne_RSVA | TCTTGAGAGGTCCAATGGATTTC | 8328-8350 |  |
| poolOne_RSVA | GATAATCAATCTCATCTTAAAGCAGRC | 8994-9020 | 895 |
| poolOne_RSVA | GCWCCTCTTAACATACTCAAACT | 9867-9889 |  |
| poolOne_RSVA | CAGATCTCAGCAAATTCAATCAAGC | 10594-10618 | 874 |
| poolOne_RSVA | GGATCACCACCACCAAATARCA | 11446-11467 |  |
| poolOne_RSVA | GAGAAATATTGAGTATGGAAAACCTAAG | 12064-12091 | 894 |
| poolOne_RSVA | GAAAATAGTCAGATATCTTATGYGCC | 12932-12957 |  |
| poolOne_RSVA | CAAATTATATCATCCYACACCWGAAAC | 13607-13633 | 895 |
| poolOne_RSVA | CCTTTAACTTACTGCCTAAGCATAC | 14478-14502 |  |
|  |  |  |  |
| poolTwo_RSVA | CWCTAACCAGAGAYATCATAACACA | 713-737 | 893 |
| poolTwo_RSVA | ATCAGGAGAGTCATGCCTG | 1587-1605 |  |
| poolTwo_RSVA | GAAAATGGTGTGATTAACTACAGTGT | 2215-2240 | 946 |
| poolTwo_RSVA | GCAGATRGATGTTTGGTTGGAT | 3139-3160 |  |
| poolTwo_RSVA | ATACCAACATACYTAAGATCYATCAG | 3739-3764 | 1014 |
| poolTwo_RSVA | GATTGAGAGTGTCCCAGGT | 4734-4752 |  |
| poolTwo_RSVA | GCAACAATCCAACYTGCTG | 5218-5236 | 860 |
| poolTwo_RSVA | CCAAGAAATCTTCTTTTCCTTTTCTT | 6052-6077 |  |
| poolTwo_RSVA | GTRTTTTGTGACACAATGAACAGT | 6754-6777 | 906 |
| poolTwo_RSVA | CCATTCAAGCAATGACCTCG | 7640-7659 |  |
| poolTwo_RSVA | GATACTACCTGACAAATATCCTTGTAG | 8180-8206 | 998 |
| poolTwo_RSVA | TTACCTCAYTWGATCGATAYTGTGT | 9153-9177 |  |
| poolTwo_RSVA | CAATGGTAGATGAAAGACAAGCC | 9793-9815 | 903 |
| poolTwo_RSVA | ACCAGGAAAATAGAGATTGTACACC | 10671-10695 |  |
| poolTwo_RSVA | AGAGGTGAAAGTCTATTATGCAGTT | 11250-11274 | 914 |
| poolTwo_RSVA | GGTGATGTAACACCAACTATATTGG | 12139-12163 |  |
| poolTwo_RSVA | GACAAAATAAGTTTGACTCAATATGTGG | 12843-12870 | 861 |
| poolTwo_RSVA | ACCTATACAATAGTCAYTCAGTGTC | 13679-13703 |  |
| poolTwo_RSVA | TGCAAGTACTGTTCYTCAGTT | 14373-14393 | 851 |
| poolTwo_RSVA | ACGAGAAAAAAAGTGTCAAAAACTAA | 15198-15223 |  |
