# Supplementary material for: Respiratory Syncytial Virus (RSV) in an Italian Pediatric Cohort: Genomic Analysis and Circulation Pattern in the Season 2022–2023
Source: J Med Virol. 2025 Oct 23;97(11):e70660. doi: 10.1002/jmv.70660 (PMC12548520; doi:10.1002/jmv.70660)
Supplement: Supplementary file 3 — Supporting Table S2: Primer pool design for RSV‐B amplification. [file JMV-97-e70660-s003.docx]

Supplementary Table S2. Primer pool design for RSV-B amplification.

| **Pool name** | **Sequence (5'→3')** | **Genome position (nt)** | **Amplified size** |
| --- | --- | --- | --- |
| poolOne_RSVB | GGGTGCAATTCACTGAGCATGA | 60-81 | 420 |
| poolOne_RSVB | TCAAGCCCAAGTAAATCAGATATTTGATTC | 451-480 |  |
| poolOne_RSVB | TGATCACAGACATGAGACCCCT | 624-645 | 397 |
| poolOne_RSVB | ACTGTTGAGCATTGTTGTTTGGA | 999-1021 |  |
| poolOne_RSVB | GTGGTATGCTATTAATCACTGAAGATGC | 1239-1266 | 653 |
| poolOne_RSVB | CCCTCCTAATTACTGCTGTAAGACC | 1628-1652 |  |
| poolOne_RSVB | GCTAAGATGGGGAGTTTTAGCCA | 1867-1889 | 417 |
| poolOne_RSVB | GCTCTACGTCATCTTCTTTGGGG | 2262-2284 |  |
| poolOne_RSVB | CCGATAACATCTGGCACCAACA | 2467-2488 | 405 |
| poolOne_RSVB | CATTGGTCATTAATGCTTCTGCTCTT | 2847-2872 |  |
| poolOne_RSVB | AAAACAGACACCAATCCATTGAATCAA | 3068-3094 | 395 |
| poolOne_RSVB | CCAGCACAGCACTTCTTGAGTT | 3442-3463 |  |
| poolOne_RSVB | ACCCCACTCATGAGATCATTGC | 3632-3653 | 410 |
| poolOne_RSVB | GTGTGCAGTTGAGGATATATGTGAAC | 4017-4042 |  |
| poolOne_RSVB | TCACACAAACCAATCCCACTCAA | 4082-4104 | 399 |
| poolOne_RSVB | TTTGATACAGCATAATGGGAGAACAC | 4456-4481 |  |
| poolOne_RSVB | GGGATCAAAAACAACATTGGGGC | 4613-4635 | 391 |
| poolOne_RSVB | GGTGATATTGTGGCTGAGTTTGTG | 4981-5004 |  |
| poolOne_RSVB | CTGGGGCAAATAACCATGGAGT | 5661-5682 | 413 |
| poolOne_RSVB | CCTCTTCTTGCTTATTGATACATTTAGGTT | 6045-6074 |  |
| poolOne_RSVB | CAAATGGGGTCAGTGTTTTAACCA | 6220-6243 | 414 |
| poolOne_RSVB | GGTGATGTGTGTAATTTCCAGCAAG | 6610-6634 |  |
| poolOne_RSVB | CTTGTAAAGTACAGTCCAATCGAGTATTT | 6745-7773 | 399 |
| poolOne_RSVB | GCATCAAACTCATCAGAAGGAAACAC | 7119-7144 |  |
| poolOne_RSVB | AGCAAAGACCAACTAAGTGGAATCA | 7353-7377 | 408 |
| poolOne_RSVB | CTTGTTTAACATGAAGTTTTGCCTCAC | 7735-7761 |  |
| poolOne_RSVB | TGTGTTGCTATGAGTAAACTTCTTATTGAG | 7912-7941 | 410 |
| poolOne_RSVB | TGGTGGTTATCTAAATGGTTGTGGT | 8298-8322 |  |
| poolOne_RSVB | GGTGTTATCTCTTTTTCAGAATGTAATGC | 8578-8605 | 402 |
| poolOne_RSVB | TGGTTGTAAGTACTGAGTTTTCATCAC | 8954-8980 |  |
| poolOne_RSVB | GGTTGCATTGTTTATCATAAAGGGCT | 9265-9290 | 399 |
| poolOne_RSVB | AAGTGTGACATACTCTTGATAGTAGATCC | 9636-9664 |  |
| poolOne_RSVB | GCTTGCAGGTGATAATAATCTCAATAACT | 9744-9772 | 411 |
| poolOne_RSVB | TTTTGGAGGAGAAATAGCTTTGTCATTT | 10128-10155 |  |
| poolOne_RSVB | GCTATCTCAACAACTCTAATCATGTGG | 10330-10356 | 411 |
| poolOne_RSVB | TTGTGACAAGAGGTATTGTTAAATGCAA | 10712-10741 |  |
| poolOne_RSVB | GATCCACAGGCTTTAGGGTCTG | 11695-11715 | 393 |
| poolOne_RSVB | ACTCTCTTTTGTCTTTGTTACAATCTAGTG | 12059-12088 |  |
| poolOne_RSVB | AGGACCTACTAAGCCATGGGTAG | 12283-12305 | 410 |
| poolOne_RSVB | AGACCAAAACTTATGCAATTTTGAAACAC | 12665-12693 |  |
| poolOne_RSVB | AAGTAACAAAGCACTTAAATCTGGATCTC | 12900-12928 | 412 |
| poolOne_RSVB | GCAAACTTGTGTCTTGATTGACTATGT | 13286-13312 |  |
| poolOne_RSVB | GGTTGTCACAGTTTTAAGTTGTGGT | 13324-13348 | 417 |
| poolOne_RSVB | CGTCATCATAGATTCAGTACTTCCACT | 13715-13741 |  |
| poolOne_RSVB | GTGATAGACAGGATTATAGATCATTCAGGT | 13840-13869 | 418 |
| poolOne_RSVB | GTTGCATCTGTAGCAGGAATGGT | 14236-14258 |  |
| poolOne_RSVB | CCTGTCACAGCCAATTGGAGTA | 14335-14356 | 412 |
| poolOne_RSVB | ACACTCTTCAATTTTGACAATGAAGTCT | 14720-14747 |  |
| poolOne_RSVB | ACAAGCTTATAAACCACAAGCATATGAA | 14804-14831 | 431 |
| poolOne_RSVB | TGTCTCGTTGTGTTGTAAATGCAC | 15206-15235 |  |
|  |  |  |  |
| poolTwo_RSVB | GACACACTGCTCTCAATTAAATGGTC | 340-366 | 413 |
| poolTwo_RSVB | GCTTGTCTTTCATCGAGTTTTCTTACA | 727-753 |  |
| poolTwo_RSVB | ACAAATATGACCTCAACCCGTAACT | 936-960 | 396 |
| poolTwo_RSVB | GTGTCTTCCCTTCCTAATCTGGAC | 1309-1332 |  |
| poolTwo_RSVB | CCAGACTGTGGGATGATAATACTGTG | 1556-1581 | 406 |
| poolTwo_RSVB | TCATAGACTTCCACAACTTGCTCC | 1939-1962 |  |
| poolTwo_RSVB | TGCAGAGCAACTCAAAGAAAATGG | 2155-2178 | 398 |
| poolTwo_RSVB | GGGTTTTCTTGGGTAGTTGGCT | 2532-2553 |  |
| poolTwo_RSVB | CATACATTAGTAGTTGCAAGTGCAGG | 2752-2777 | 401 |
| poolTwo_RSVB | TTGGTTGATTGATTGGTTGGTGG | 3131-3153 |  |
| poolTwo_RSVB | GCAAGCATCAACATACTAGTAAAGCA | 3376-3401 | 406 |
| poolTwo_RSVB | GCATTTTTGAATTCGGTGGTTGC | 3760-3782 |  |
| poolTwo_RSVB | GAGTCAATTTATAGTGGATCTTGGTGC | 3876-3902 | 408 |
| poolTwo_RSVB | TCTATTGTGATGGATGTGTTTCCCA | 4260-4284 |  |
| poolTwo_RSVB | GCAAATTTTGGCCCTATTTTACACTAATAC | 4293-4322 | 405 |
| poolTwo_RSVB | TCCCAGGTCTTTTCTAGAGTCCTG | 4675-4698 |  |
| poolTwo_RSVB | CACAAAGTTACACTAACAACTGTCACA | 4844-4870 | 415 |
| poolTwo_RSVB | GGTGGTTTGTTTGTGGGTTTTGT | 5237-5259 |  |
| poolTwo_RSVB | ACACACCAGCTGTCAACAACC | 5971-5991 | 408 |
| poolTwo_RSVB | CTGGTGATTTCCAACAATCTGCTG | 6356-6379 |  |
| poolTwo_RSVB | GTAAGGCAACAAAGTTATTCTATCATGTCT | 6516-6545 | 407 |
| poolTwo_RSVB | NCATGACACTATAGCTCCTAGAGAAG | 6898-6923 |  |
| poolTwo_RSVB | ATGTCAACAAGCTGGAAGGCAA | 7048-7069 | 412 |
| poolTwo_RSVB | GTTGATTTGGGATTGATGGTCAGC | 7036-7460 |  |
| poolTwo_RSVB | CATTGCTTGAATGGTAGAAGATGCC | 7666-7690 | 404 |
| poolTwo_RSVB | CAGATGGATGGTTTGCTTGCTG | 8049-8070 |  |
| poolTwo_RSVB | ACCACAACCATTTAGATAACCACCA | 8296-8322 | 399 |
| poolTwo_RSVB | TCATATGCTCTATTAGTGGGCTTTGT | 8670-8695 |  |
| poolTwo_RSVB | TGTCCTCGTCTGAACAAATTGCT | 8807-8829 | 403 |
| poolTwo_RSVB | AAACCCATGACTTTTTACCTCATTTGA | 9184-9210 |  |
| poolTwo_RSVB | TGCTATCACAATTATTCCTTTACGGAGA | 9448-9470 | 411 |
| poolTwo_RSVB | TGCAGTTAATTCTTACAGCATCCATTG | 9833-9859 |  |
| poolTwo_RSVB | TGATTTGATTATTTTATCAGGATTGCGGT | 10050-10078 | 401 |
| poolTwo_RSVB | TTTTTCTGCTAAGATTTGGATTTGCCT | 10425-10451 |  |
| poolTwo_RSVB | AATGAAGTTGATGAACAAAGTGGGTT | 10796-10821 | 414 |
| poolTwo_RSVB | AGTATTGTATTTATCCATGGACCTACTCTC | 11179-11210 |  |
| poolTwo_RSVB | AAAAACATCAGCGATAGATACAACTGA | 11971-11997 | 407 |
| poolTwo_RSVB | TGGTCTCTTTGCTTTTTGGTTAAAACT | 12352-12378 |  |
| poolTwo_RSVB | CACCGGTTAACAGTCAGTAGTAGAC | 12529-12551 | 400 |
| poolTwo_RSVB | TGAGATCCAGATTTAAGTGCTTTGTTAC | 12902-12929 |  |
| poolTwo_RSVB | GCTGGACATTGGATTCTGATTATTCAA | 13060-13032 | 346 |
| poolTwo_RSVB | TCTATGTTAACAACCCAAGGGCA | 13384-13406 |  |
| poolTwo_RSVB | AGTTACAACTTTTCAGACAACACTCA | 13544-13571 | 415 |
| poolTwo_RSVB | AGGAAGCATGCAATAAAGTGATGC | 13934-13959 |  |
| poolTwo_RSVB | CCCAGTTGTATAGCATTCATAGGTGA | 14053-14076 | 413 |
| poolTwo_RSVB | TTTGAAATCAATATCATCTTGAGCATGGT | 14438-14466 |  |
| poolTwo_RSVB | ACTTCCTGTTTTTGATGTTGTGCAA | 14565-14589 | 402 |
| poolTwo_RSVB | CTTCTTGAGCTCATTGGTTGTTAAACT | 14941-14967 |  |
